# Editorial: New basic and translational perspectives on skin repair

**DOI:** 10.3389/fphys.2024.1469900

**Published:** 2024-09-17

**Authors:** Yujie Li, Jibin Xia, Yiming Zhang

**Affiliations:** ^1^ Department of Plastic and Cosmetic Surgery, Xinqiao Hospital, Army Medical University, Chongqing, China; ^2^ Department of Administration, Army 73rd Group Military Hospital, Xiamen, China

**Keywords:** skin repair, wound healing, cellular senescence, inflammation, angiogenesis, SCAR

## Introduction

Skin repair remains a critically important clinical challenge, one that has been the focus of extensive research for over a century. Our understanding of the cellular and molecular underpinnings of the intricate interactions that facilitate cellular and tissue movement during wound repair has significantly advanced. Wound healing is a dynamic and intricate biological process characterized by a high degree of order, coordination, and interactivity among various tissues and cell types. It necessitates the precise orchestration of inflammation, angiogenesis, cell migration, proliferation, matrix deposition, and remodeling under stringent regulatory control. Despite substantial progress in deciphering the molecular mechanisms of wound healing, challenges persist, particularly in addressing complex conditions like diabetic foot ulcers, chronic non-healing wounds, and hypertrophic scars. The current advancements have yet to yield fully effective treatments for these stubborn clinical problems. It is startling to note that since the U.S. Food and Drug Administration (FDA) approved tissue-engineered skin as a therapeutic approach for wound healing in 1997, no new therapeutic candidate (excluding physical therapies, devices, dressings, and antimicrobial agents) has been approved for clinical application.

The Research Topic consists of 3 original Research and 7 reviews, collectively examining the impact of skin structure, senescence, inflammation, and angiogenesis on the pathophysiology of wound healing. It also explores novel therapeutic approaches. It is essential to emphasize that the Research Topic is focused on skin repair rather than skin regeneration. Skin Repair typically results in scar formation without appendages, while skin regeneration aims to restore the skin with functional appendages, which is rare in adult mammals. In general, these studies synthesize recent advancements and propose innovative perspectives and concepts aimed at propelling future wound healing research.

## Key factors of skin repair

Dermal adipocytes are integral to the wound healing process, with the dermal white adipose tissue (dWAT), a recently identified adipocyte layer within the reticular dermis, exhibiting notable plasticity and adaptability surpassing that of other adipose tissues. A review by Li et al. has delineated the proposed roles of dWAT in wound healing, highlighting its capacity to modulate inflammatory responses, trans differentiation into fibroblasts, stimulate extracellular matrix (ECM) synthesis, and produce antimicrobial peptides upon skin injury. Concurrently, the epidermal permeability barrier, essential for both dermal and extra-dermal functions, can be rapidly restored following damage through the topical application of natural compounds, as outlined by Lei et al. This restoration is facilitated through processes including keratinocyte differentiation, enhanced lipid and hyaluronic acid synthesis, antioxidant activity, and the upregulation of aquaporin-3 and sodium-hydrogen exchange protein 1, thereby fortifying the skin’s osmotic defense.

Cellular senescence is a biological process that inhibits abnormal cell proliferation during tissue repair. However, an excessive accumulation of senescent cells can lead to chronic inflammation, tissue dysfunction, and the development of refractory wounds. Kita et al. have reviewed the distinct roles of cellular senescence in the context of wound healing, diabetic skin, and skin aging. During physiological wound healing, senescent cells facilitate extracellular matrix deposition and contribute to tissue fibrosis. The buildup of senescent fibroblasts, melanocytes, and keratinocytes is associated with the manifestation of aging characteristics in the skin. Furthermore, cellular senescence plays a role in the pathogenesis of diabetic ulcers. Liu et al. have demonstrated that aging affects neutrophil function, exacerbates immune dysregulation, and consequently delays wound closure.

Chronic wounds are characterized by persistent inflammation, a condition often exacerbated by factors such as bacterial colonization, diabetes mellitus, and lupus erythematosus, leading to the prolonged presence of immune cells within the wound and impeding the healing process. Researchers Zhang et al. have developed a rapidly cross-linkable hydrogel with potent antibacterial capabilities and superior biocompatibility. This hydrogel demonstrates effective contact-killing activity against both Gram-negative bacteria, such as *Escherichia coli*, and Gram-positive bacteria, such as *Staphylococcus aureus*, and also prevents biofilm formation. In a separate study, Wu et al. identified 41 differentially expressed genes (DEGs) shared between diabetic foot ulcers (DFUs) and cutaneous lupus erythematosus (CLE) through transcriptomic analysis. Their findings indicate that both conditions are associated with epidermal cell abnormalities and inflammatory responses, revealing a molecular commonality and a significant link between DFU and CLE.

Neovascularization has emerged as a pivotal area of interest in wound healing research. The development of new vascular networks is integral to every phase of the healing process, with the complex interplay between angiogenesis and the inflammatory response—highlighted by immune cell involvement and cytokine collaboration—forming a fundamental component of tissue repair. Shi et al. have synthesized the regulatory mechanisms governing blood vessel formation within the wound healing context, detailing the dynamics of endothelial cell proliferation, migration, and the secretion of angiogenic factors across various healing scenarios. Furthermore, their research delves into the subtle yet significant relationship between the inflammatory milieu and angiogenesis during wound healing.

## Scarring

Scarring is a significant challenge that arises from wounds. To develop effective treatments that prevent abnormal scar formation, such as keloids and hypertrophic scars, a deeper understanding of the cellular and molecular mechanisms involved is essential. Hong et al. have extensively reviewed strategies for reducing thyroidectomy scars in the early postoperative period. Their findings indicate that meticulous surgical incision suturing and the selection of appropriate suture materials are crucial for scar prevention. Timely intervention is imperative for managing hypertrophic scars post-thyroidectomy, with potential treatments including local botulinum toxin injections, steroid administration, or the use of tension-reducing devices. Qiu et al. have evaluated the utility of negative pressure wound therapy (NPWT) as an adjunctive treatment in scar management. NPWT, a physical therapy method, has been shown to alleviate wound tension, stabilize grafts, and enhance the quality of wound beds. Furthermore, it promotes microcirculation, lymphatic drainage, granulation tissue development, and the removal of exudate and necrotic tissue. Cai et al. have introduced an innovative approach combining adipose tissue extract (ATE) with fractional laser therapy for hypertrophic scar treatment. This method has been demonstrated to decrease inflammatory infiltration, reduce α-SMA expression, and consequently lead to a reduction in scar volume, improved texture, and a thinner dermis.

The overarching objective of wound healing research is to comprehensively elucidate the contributions of various cell lineages to the wound healing process and to decipher the underlying molecular mechanisms and biochemical signaling pathways. This knowledge is crucial for the advancement of innovative therapeutic strategies. The Research Topic provides some recent advancements in our understanding of the fundamental processes involved in skin repair and highlights several pioneering treatment approaches. It is anticipated that this work will offer fresh insights, fostering further fundamental, preclinical, and clinical investigations aimed at fulfilling the existing gaps in skin repair therapies.

